# Genetic and Morphological Evidence Implies Existence of Two Sympatric Species in *Cyathopharynx furcifer* (Teleostei: Cichlidae) from Lake Tanganyika

**DOI:** 10.1155/2012/980879

**Published:** 2012-05-20

**Authors:** Tetsumi Takahashi, Michio Hori

**Affiliations:** Laboratory of Animal Ecology, Graduate School of Science, Kyoto University, Kitashirakawa-Oiwake, Sakyo, Kyoto 606-8502, Japan

## Abstract

Although the cichlid fishes from Lake Tanganyika are treated as a textbook example of adaptive radiation, many taxonomic problems remain unresolved. *Cyathopharynx furcifer*, which belongs to the currently monospecific genus *Cyathopharynx*, contains two colour morphs at the southern end of the lake: one has a yellow anal fin, and the other has a black anal fin. Some books for hobbyists of ornamental fish treat these morphs as different species, but taxonomic studies have neither mentioned the existence nor addressed the status of these colour morphs. In the present paper, we analysed these two colour morphs using mitochondrial, microsatellite, morphometric, and meristic data sets. Both molecular and morphological data allowed clear discrimination between these morphs, suggesting the existence of two distinct sympatric species. Three taxonomic species have been described in this genus, and only *C. furcifer* is currently considered valid. Observations of type specimens of these three nominal species will be needed to determine the scientific names of these colour morphs.

## 1. Introduction

Lake Tanganyika is one of the ancient lakes of the East African Rift Valley. This lake harbours about 250 cichlid species, and 98% of these species are endemic to the lake [1]. These fish exhibit high morphological, behavioural, ecological, and genetical diversification, and are treated as a textbook example of adaptive radiation (e.g., [2–7]).

*Cyathopharynx* Regan is one of the genera belonging to the endemic tribe Ectodini from Lake Tanganyika [8, 9]. This genus is morphologically well defined, namely, fish of this genus have small scales on the sides of the body (48–64 scales in longitudinal line), a lower pharyngeal bone with a rounded posterior margin, and in males, long pelvic fins. These morphological features are also found in some other genera of Ectodini [8], but only *Cyathopharynx* has all of these features combined. A phylogenetic study based on mitochondrial DNA does not contradict the monophyly of *Cyathopharynx* and shows that this genus nest within a monophyletic group including *Ophthalmotilapia* Pellegrin and *Cardiopharynx* Poll [10]. Three species have been described in *Cyathopharynx*: *C. furcifer *(Boulenger) (originally described as *Paratilapia furcifer* in 1898 [11]), *C. foae* (Vaillant) (originally described as *Ectodus foae* in 1899 [12]), and *C. grandoculis* (Boulenger) (originally described as *Tilapia grandoculis* in 1899 [13]). The latter two nominal names are currently considered as junior synonyms of *C. furcifer*, and only *C. furcifer *is considered valid in this genus [8, 14].

*Cyathopharynx furcifer* is a common species in rocky shorelines of the lake and exhibits sexual dimorphism: males have a colourful, iridescent body, and elongated pelvic fins, whereas females are not colourful and their pelvic fins are moderate in length. This fish is a maternal mouth-brooder. Mature males build mating craters on the sandy lake bottom or on the flat surface of a large stone, to which they attract females. Females deposit eggs in the crater, and pick them up into their mouths before leaving the crater [15–17]. The function of the craters is not well known, but the size and neatness of craters may provide conspecifics with information about the owner's size, capability, and condition [17].

**Figure 1:**
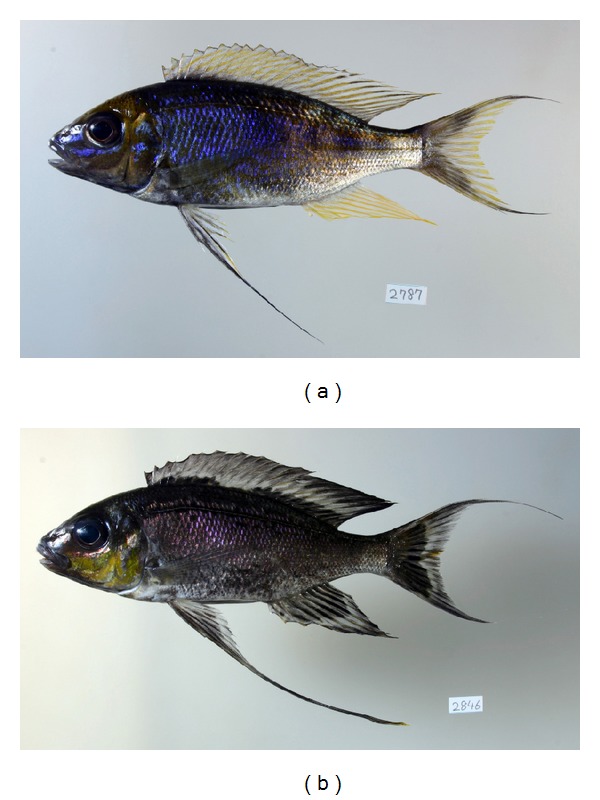
Two colour morphs of *Cyathopharynx furcifer*. (a) YA, male, 127.8 mm SL. (b) BA, male, 114.4 mm SL.

At Kasenga at the southern end of the lake, two colour morphs exist in males of *C. furcifer* (Figure 1). One morph has a bluish body, orange forehead, and a yellow anal fin (hereafter YA, which means yellow-anal-fin morph), while the other morph has a blackish body, orange cheeks, and a black anal fin (hereafter BA, which means black-anal-fin morph). No males with intermediate or mixed colour patterns between the morphs have been found. Some books for hobbyists of ornamental fish treat YA as *C. furcifer* because the body colouration of this morph accords with that of the type specimens of *C. furcifer*, and BA as *C. foae *(or *C. foai*) without any distinct reason [18]. However, taxonomic studies have neither mentioned the existence nor addressed the status of these sympatric colour morphs. In the present study, molecular and morphological analyses were conducted to test whether these sympatric morphs are different species.

## 2. Methods

### 2.1. Fish Samples

Fish were collected at Kasenga near Mpulungu, Zambia, at the southern end of Lake Tanganyika, with a screen net in November and December 2006. The right pectoral fins of the fish were fixed in 100% ethanol for DNA extraction. The bodies of the fish were fixed in 10% formalin and preserved in 50% isopropyl alcohol for morphological examination. The sex of the fish was determined from the shape of the genital papilla. Only large males with fully expressed body colour were used for molecular and morphological analyses in order to avoid misidentification of morphs (*N* = 32, 100.7–137.3 mm standard length (SL) in YA, *N* = 32, 121.5–138.8 mm SL in BA).

### 2.2. DNA Extraction and Amplification

Total DNA was extracted using an AquaPure Genomic DNA Kit (Bio-Rad). Polymerase chain reaction (PCR) was conducted using a PC 818 Program Temp Control System (Astec) for the amplification of the mitochondrial DNA (mtDNA) and the microsatellite loci using the following programme: one cycle of 94°C for 2 min; 30 cycles of 94°C for 15 s, annealing temperature specific to each primer set for 15 s, 72°C for 30 s; one cycle of 72°C for 7 min.

A partial mtDNA sequence, including a portion of cyt *b* (1125 bp), was amplified with the primers H15915 [19] and L14724 [20] (annealing temperature 53°C). The PCR fragments of the mtDNA were purified using the ExoSAPIT enzyme mix (USB), directly sequenced with BigDye sequencing chemistry (Applied Biosystems), and analysed on an ABI 3130xl sequencer (Applied Biosystems). Sequences are available in the DNA Data Bank of Japan (DDBJ Accession no. AB691241–AB691304).

Five microsatellite loci were used for genotyping: GM264 [21], Pzeb4 [22], Ttem8 and Ttem9′ [23], and UNH2050 [24] (annealing temperature 55°C). Forward primers were labelled with florescent dye NED (GM264), HEX (Pzeb4, UNH2050), or 6-FAM (Ttem8, Ttem9′). The microsatellite loci were analysed on an ABI 3130xl Sequencer using internal size marker Genescan 400 HD (Applied Biosystems).

### 2.3. Analyses of Molecular Data

For the mtDNA sequences, a haplotype network was constructed from the maximum-likelihood (ML) and maximum parsimonious (MP) trees, which were translated into maximum parsimony branch lengths in PAUP* version 4.0b10 [25]. The ML tree was generated based on the HKY model selected by hierarchical likelihood ratio tests implemented in ModelTest 3.5 [26].

Departure from Hardy-Weinberg (HW) equilibrium for every microsatellite locus and linkage disequilibrium (LD) for all pairs of loci were tested within each of the two morphs using Arlequin version 3.11 [27] (100 000 steps in the Markov chain, 1000 dememorization steps in the HW test; 10 000 permutations in the LD test). Critical significance levels were corrected following the sequential Bonferroni procedure [28]. A Bayesian model-based clustering algorithm was implemented in Structure 2.3.3 [29] to test the assignment of *K* ancestors with admixture and independent allele frequency models (100 000 iterations were run after an initial burn-in period of 50 000 iterations). *K* was set from 1 to 5, and 10 independent runs were performed for each *K*. The value of *K* = 2 was chosen, which showed the highest Δ*K* [30].

Genetic differentiation between the morphs was assessed by analyses of molecular variance (AMOVA) for both mtDNA and microsatellite data as implemented in GENALEX version 6.41 [31]. Genetic significance tests between morphs were conducted using 9999 permutations.

### 2.4. Morphological Data

Methods for measuring 13 morphometric characters (SL, body depth, length and width of head, snout length, eye length, interorbital width, lower jaw length, length and depth of caudal peduncle, dorsal fin base length, anal fin base length, and pelvic fin length) and counting 9 meristic characters (numbers of spines and soft rays in dorsal fin, number of anal fin soft rays, number of pectoral fin soft rays, number of scales in longitudinal line, numbers of scales on upper and lower lateral lines, number of gill rakers on lower limb of the most rostral gill-arch, and number of outer teeth on premaxillae) correspond with those of Snoeks [32], except for pelvic fin length, which was measured from the base to the tip of the longest ray. Measurements were taken to the nearest 0.1 mm using dividers or digital callipers under a binocular microscope. The last two soft rays of dorsal and anal fins were counted as two soft rays, although those are sometimes counted as one soft ray in noncichlid fishes (i.e., [33]).

### 2.5. Analyses of Morphological Data

The 13 morphometric characters were log_10_ transformed. Twelve morphometric characters except for SL were analysed by the multivariate analysis of covariance (MANCOVA) with SL as covariate. The nine meristic characters were analysed by the multivariate analysis of variance (MANOVA, note that body size was not considered in this analysis because the meristic characters were not significantly correlated with SL  : *F*_9,53_ = 0.601, *P* = 0.791). When the significant differences were found in these analyses, the analyses of covariance (ANCOVAs) with log_10_ transformed SL as covariate for the 12 log_10_ transformed morphometric characters and the analyses of variance (ANOVAs) for the 9 meristic characters were carried out in order to suggest which character was different between morphs. Critical significance levels were corrected following the sequential Bonferroni procedure [28].

The linear discriminant analyses (LDAs) were carried out in order to visualize the degrees of morphological differences between morphs. In the LDA based on the morphometric characters, each measured value was standardized with SL using the following formula:

(1) $$\;Y_{ij}^{\prime }=\;\log\left(Y_{ij}\right)-a_{j}\log\left(L_{i}\right),$$

where *Y*_*ij*_′ and *Y*_*ij*_ are the standardized and raw values of character *j* of individual *i*, respectively, *a*_*j*_ is the pooled regression coefficient of character *j* for the two morphs, and *L*_*i*_ is the SL of individual *i*. The LDA for the meristic characters was conducted based on the raw data.

## 3. Results

### 3.1. Analyses of mtDNA Sequences

**Figure 2:**
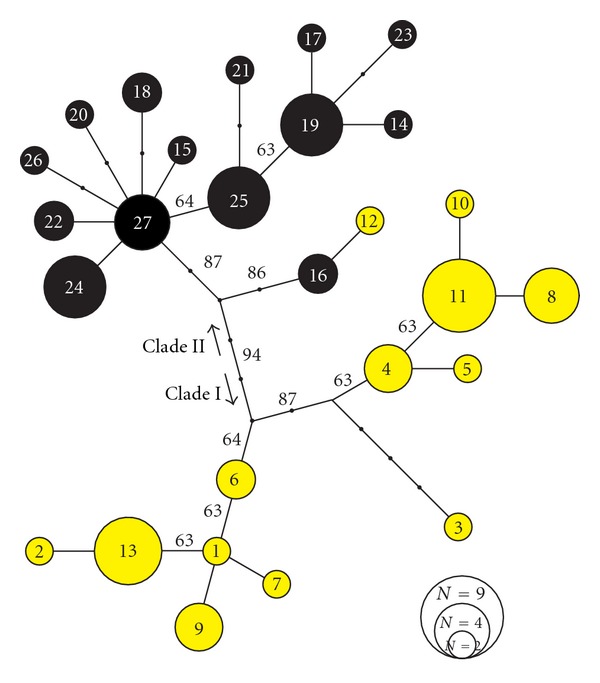
Unrooted haplotype network based on mtDNA sequences. Haplotypes are numbered from 1 to 27 and coloured according to morph (yellow circles: YA, black circles: BA). The size of circles reflects the number of specimens sharing the same haplotype (see explanation in the lower right corner). Only bootstrap values >50% are shown.

A total of 27 mtDNA haplotypes was obtained in the 64 individuals. Proportion of the variance of genetic diversity between the two morphs was significantly larger than zero (AMOVA: degree of freedom = 1, proportion of variance between the morphs = 0.089, *P* < 0.001). The ML tree separated the 64 individuals into two clusters (Figure 2). One cluster consists of 31 out of the 32 individuals of YA (clade I), and the remaining 1 individual of YA and the 32 individuals of BA formed the other cluster (clade II). The separation of these two clusters was supported by a 94% bootstrap probability. One MP tree was obtained (CI = 0.976, RC = 0.966), which accorded with the ML tree in topology.

### 3.2. Analyses of Microsatellite Allele Frequencies

**Figure 3:**
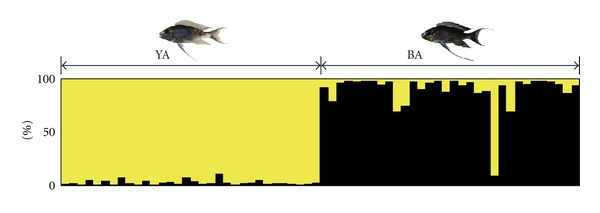
Results of the population assignment test based on five microsatellite loci.

Based on the microsatellite data, no LD was found in any of the possible pairs among the five markers in the two morphs (likelihood ratio tests: *P* > 0.05 in 20 tests after sequential Bonferroni correction). Allele frequencies showed no significant departures from HW equilibrium (Table 1). Proportion of the variance of genetic diversity between the two morphs was significantly larger than zero (AMOVA: degree of freedom = 1, proportion of variance between the morphs = 0.190, *P* < 0.001). A Bayesian population assignment test to the two groups indicated that the 32 individuals of YA and 1 individual of BA were clustered together, and the remaining 31 individuals of BA formed the other cluster (Figure 3). The BA individual that was clustered in YA group in the microsatellite data was included in the clade II of the mitochondrial tree (haplotype no. 19, Figure 2).

### 3.3. Analyses of Morphological Characters

**Figure 4:**
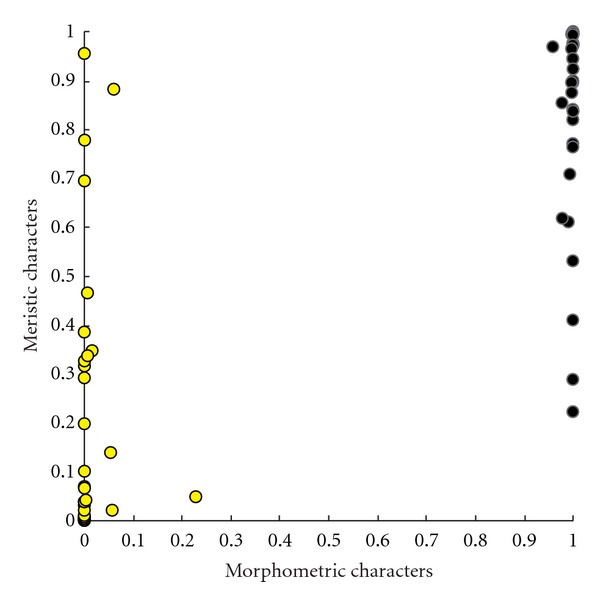
Plot of probabilities that an individual is BA estimated by the linear discriminant analyses of morphometric and meristic characters. Yellow circles indicate YA males, and black circles indicate BA males.

The MANCOVA for morphometric characters and the MANOVA for meristic characters revealed significant morphological differences between colour morphs (Tables 2 and 3). The ANCOVAs for morphometric characters and the ANOVAs for meristic characters revealed that YA had significantly smaller head, smaller eyes, shorter pelvic fins, and smaller number of gill rakers than BA did, although the ranges of these characters largely overlapped between morphs (e.g., 14–16 gill rakers in YA, whereas15–18 gill rakers in BA). In the LDAs (Figure 4), the morphometric characters more clearly discriminated the morphs (error rate was 0.0%) than the meristic characters did (error rate was 10.9%).

## 4. Discussion

The present genetic analyses based on mtDNA sequences and microsatellites revealed that the gene flow is restricted between two colour morphs of *C. furcifer*. At Kasenga, males of these morphs build nests side by side on the lake bottom, and spatial and temporal barriers that would cause reproductive isolation between morphs are not found. Assortative mating by mate choice seems most likely to cause reproductive isolation between the morphs. These morphs were also different in morphological characters, supporting the idea that these morphs are distinct sympatric species. Some females have a yellowish anal fin and some other females have a blackish anal fin. These females may correspond to YA and BA, respectively. However, the colours of the anal fins of females are paler than those of large males, and it is difficult to determine the colours of the anal fins in some females. Molecular and morphological analyses will be useful to determine the morphs of females and small males, as the present data showed clear discrimination between the morphs in large males. In this study, one large male of YA and one large male of BA exhibited discrepancies in clustering between their mitochondrial and microsatellite data. This may have been caused by insufficient molecular data, by incomplete lineage sorting, or by hybridization between the morphs. In cichlid fish from Lake Tanganyika, incomplete lineage sorting is reported among tribes [34], and hybridization is reported between populations, between species, and between genera as a means by which rapid diversification can be achieved [35–42].

Boulenger published a description of *Cyathopharynx furcifer* on December 1898 [43]. This is the first full description of this species, but not the original description. Boulenger published a synopsis of this full description on June 1898 [11]. This short synopsis is the original description of this species because it was published earlier than the full description [14], although only a few morphological features are described. According to the full description, two syntypes of this species from Kinyamkolo, close to the present sampling locality, Kasenga, have elongated pelvic fins, bluish dorsal part and white ventral part of the body, some yellow marbling on the postocular part of the head, and some yellow streaks on the dorsal and anal fins [43]. These features accord with those of large males of YA (Figure 1), as some books for hobbyists of ornamental fish pointed out [18].

Although taxonomic studies currently treat *Cyathopharynx foae* and *C. grandoculis* as junior synonyms of *C. furcifer *[8, 14], some books for hobbyists of ornamental fish treat *C. foae* as a valid species that corresponds to BA, and *C. grandoculis *as a junior synonym of *C. foae* [18]. The taxonomic status of these two nominal species (*C. foae *and *C. grandoculis*) has not been tested with taking sexual and developmental variations into account (e.g., [44]). The holotypes of these two nominal species appear to be small males or females, as indicated by the small body size in *C. foae* (64 mm SL [12]) and short pelvic fins in *C. grandoculis* [13]. Morphological analyses, and if possible, molecular analyses, of type specimens of the three nominal species, and comparisons of these type specimens with nontype specimens of various body sizes, localities, and sexes will be needed to determine which nominal species corresponds to YA or BA, or possibly even to a yet undescribed species.

## Figures and Tables

**Table 1:** Details of microsatellite loci of the 72 large adults that are genotyped in the present study. (*H*_*o*_: observed heterozygosity, *H*_*e*_: expected heterozygosity, ^NS^ *P* > 0.05 in a test of departure from Hardy-Weinberg equilibrium after a sequential Bonferroni correction).

	*N*	No. of alleles	*H*_*o*_	*H*_*e*_
YA				
GM264	32	7	0.750^NS^	0.743
Pzeb4	32	11	0.750^NS^	0.789
Ttem8	32	7	0.656^NS^	0.600
Ttem9′	32	12	0.875^NS^	0.820
UNH2050	32	8	0.469^NS^	0.605

BA				
GM264	32	21	1.000^NS^	0.930
Pzeb4	32	14	0.750^NS^	0.879
Ttem8	32	16	0.750^NS^	0.887
Ttem9′	32	14	0.906^NS^	0.853
UNH2050	32	11	0.781^NS^	0.743

**Table 2:** Differences in log_10_ transformed morphometric characters between adult males of YA and BA (**P ≤ 0.01, *P ≤ 0.05, ^NS^ *P* > 0.05 after a sequential Bonferroni correction).

	Morphs	log_10_ SL	Morph × log_10_ SL
MANCOVA	*F*_12,49_ = 7.79**	*F*_12,49_ = 77.5**	*F*_12,49_ = 1.46^NS^
ANCOVAs			
Body depth	*F*_1,60_ = 6.32^NS^	*F*_1,60_ = 97.2**	*F*_1,60_ = 0.0203^NS^
Head length	*F*_1,60_ = 12.0**	*F*_1,60_ = 146**	*F*_1,60_ = 2.76^NS^
Head width	*F*_1,60_ = 1.18^NS^	*F*_1,60_ = 71.9**	*F*_1,60_ = 0.266^NS^
Snout length	*F*_1,60_ = 1.99^NS^	*F*_1,60_ = 134**	*F*_1,60_ = 1.96^NS^
Eye length	*F*_1,60_ = 48.7**	*F*_1,60_ = 21.1**	*F*_1,60_ = 2.25^NS^
Interorbital width	*F*_1,60_ = 0.0484^NS^	*F*_1,60_ = 51.1**	*F*_1,60_ = 0.454^NS^
Lower jaw length	*F*_1,60_ = 5.77^NS^	*F*_1,60_ = 32.5**	*F*_1,60_ = 1.59^NS^
Caudal peduncle length	*F*_1,60_ = 6.11^NS^	*F*_1,60_ = 50.6**	*F*_1,60_ = 2.24^NS^
Caudal peduncle depth	*F*_1,60_ = 1.10^NS^	*F*_1,60_ = 110**	*F*_1,60_ = 1.04^NS^
Dorsal fin base length	*F*_1,60_ = 8.21^NS^	*F*_1,60_ = 460**	*F*_1,60_ = 1.37^NS^
Anal fin base length	*F*_1,60_ = 2.90^NS^	*F*_1,60_ = 90.0**	*F*_1,60_ = 0.0411^NS^
Pelvic fin length	*F*_1,60_ = 12.8**	*F*_1,60_ = 17.4**	*F*_1,60_ = 4.08^NS^

**Table 3:** Differences in meristic characters between adult males of YA and BA (**P ≤ 0.01, *P ≤ 0.05, ^NS^ *P* > 0.05 after a sequential Bonferroni correction).

MANOVA	*F*_9,54_ = 7.70**
ANOVAs	
Dorsal fin spines	*F*_1,62_ = 0.984^NS^
Dorsal fin soft rays	*F*_1,62_ = 8.12*
Anal fin soft rays	*F*_1,62_ = 2.00^NS^
Pectoral fin rays	*F*_1,62_ = 0.463^NS^
Scales in longitudinal line	*F*_1,62_ = 4.67^NS^
Scales on upper lateral line	*F*_1,62_ = 8.27*
Scales on lower lateral line	*F*_1,62_ = 1.16^NS^
Gill rakers	*F*_1,62_ = 33.5**
Outer teeth on premaxillae	*F*_1,62_ = 10.0*

## References

[1] Snoeks J (2000). How well known is the ichthyodiversity of the large East African lakes?. *Advances in Ecological Research*.

[2] Turner GF, Seehausen O, Knight ME, Allender CJ, Robinson RL (2001). How many species of cichlid fishes are there in African lakes?. *Molecular Ecology*.

[3] Turner GF (2007). Adaptive radiation of cichlid fish. *Current Biology*.

[4] Koblmüller S, Sefc KM, Sturmbauer C (2008). The Lake Tanganyika cichlid species assemblage: recent advances in molecular phylogenetics. *Hydrobiologia*.

[5] Salzburger W (2009). The interaction of sexually and naturally selected traits in the adaptive radiations of cichlid fishes. *Molecular Ecology*.

[6] Sefc KM (2011). Mating and parental care in Lake Tanganyika’s cichlids. *International Journal of Evolutionary Biology*.

[7] Takahashi T, Koblmüller S (2011). The adaptive radiation of cichlid fish in Lake Tanganyika: a morphological perspective. *International Journal of Evolutionary Biology*.

[8] Poll M (1986). Classification des cichlidae du Lac Tanganika. tribus, genres et espèces. *Académie Royale de Belgique Mémoires de la Classe des Sciences*.

[9] Takahashi T (2003). Systematics of Tanganyikan fishes (Teleostei: Perciformes). *Ichthyological Research*.

[10] Koblmüller S, Salzburger W, Sturmbauer C (2004). Evolutionary relationships in the sand-dwelling cichlid lineage of Lake Tanganyika suggest multiple colonization of rocky habitats and convergent origin of biparental mouthbrooding. *Journal of Molecular Evolution*.

[11] Boulenger GA (1898). Report on the fishes recently obtained by Mr. J.E.S. Moore in Lake Tanganyika. *Proceedings of the Zoological Society of London*.

[12] Vaillant ML (1899). *Protopterus retropinnis* et *Ectodus Foae*, espèces nouvelles de l’Afrique équatoriale. *Bulletin du Muséum National d’Histoire Naturelle*.

[13] Boulenger GA (1899). Second contribution to the ichthyology of Lake Tanganyika. on the fishes obtained by the Congo Free State Expedition under Lieut. Lemaire in 1898. *Transaction of the Zoological Society of London*.

[14] Maréchal C, Poll M, Daget J, Gosse JP, Teugels GG, Thys van den Audenaerde DFE (1991). Cyathopharynx. *Check-List of the Freshwater Fishes of Africa*.

[15] Karino K (1997). Female mate preference for males having long and symmetric fins in the bower-holding cichlid *Cyathopharynx furcifer*. *Ethology*.

[16] Rossiter A, Yamagishi S, Kawanabe H, Hori M, Nagoshi M (1997). Intraspecific plasticity in the social system and mating behaviour of a lek-breeding cichlid fish. *Fish Communities in Lake Tanganyika*.

[17] Schaedelin FC, Taborsky M (2006). Mating craters of *Cyathopharynx furcifer* (cichlidae) are individually specific, extended phenotypes. *Animal Behaviour*.

[18] Konings A (1998). *Tanganyika Cichlids in Their Natural Habitat*.

[19] Edwards SV, Wilson AC (1990). Phylogenetically informative length polymorphism and sequence variability in mitochondrial DNA of Australian songbirds (*Pomatostomus*). *Genetics*.

[20] Palumbi SR, Martin A, Romano S, McMillian WO, Stice L (1991). *The Simple Fool’s Guide to PCR*.

[21] Streelman JT, Albertson RC, Kocher TD (2003). Genome mapping of the orange blotch colour pattern in cichlid fishes. *Molecular Ecology*.

[22] van Oppen MJH, Rico C, Deutsch JC, Turner GF, Hewitt GM (1997). Isolation and characterization of microsatellite loci in the cichlid fish *Pseudotropheus zebra*. *Molecular Ecology*.

[23] Takahashi T, Watanabe K, Munehara H, Rüber L, Hori M (2009). Evidence for divergent natural selection of a Lake Tanganyika cichlid inferred from repeated radiations in body size. *Molecular Ecology*.

[24] Albertson RC, Streelman JT, Kocher TD (2003). Directional selection has shaped the oral jaws of Lake Malawi cichlid fishes. *Proceedings of the National Academy of Sciences of the United States of America*.

[25] Swofford DL (2002). *PAUP: Phylogenetic Analysis Using Parsimony (and Other Methods)*.

[26] Posada D, Crandall KA (1998). ModelTest: testing the model of DNA substitution. *Bioinformatics*.

[27] Excoffier L, Laval G, Schneider S (2005). Arlequin, (version 3.0): an integrated software package for population genetics data analysis. *Evolutionary Bioinformatics Online*.

[28] Rice WR (1989). Analyzing tables of statistical tests. *Evolution*.

[29] Pritchard JK, Stephens M, Donnelly P (2000). Inference of population structure from multilocus genotype data. *Genetics*.

[30] Evanno G, Regnaut S, Goudet J (2005). Detecting the number of clusters of individuals using the software structure: a simulation study. *Molecular Ecology*.

[31] Peakall R, Smouse PE (2006). GENALEX 6: genetic analysis in Excel. Population genetic software for teaching and research. *Molecular Ecology Notes*.

[32] Snoeks J, Snoeks J (2004). Material and methods. *The Cichlid Diversity of Lake Malawi/Nyasa/Niassa: Identification, Distribution and Taxonomy*.

[33] Hubbs CL, Lagler KF (2004). *Fishes of the Great Lakes Region*.

[34] Takahashi K, Terai Y, Nishida M, Okada N (2001). Phylogenetic relationships and ancient incomplete lineage sorting among cichlid fishes in lake Tanganyika as revealed by analysis of the insertion of retroposons. *Molecular Biology and Evolution*.

[35] Rüber L, Meyer A, Sturmbauer C, Verheyen E (2001). Population structure in two sympatric species of the Lake Tanganyika cichlid tribe Eretmodini: evidence for introgression. *Molecular Ecology*.

[36] Salzburger W, Baric S, Sturmbauer C (2002). Speciation via introgressive hybridization in East African cichlids?. *Molecular Ecology*.

[37] Schelly R, Salzburger W, Koblmüller S, Duftner N, Sturmbauer C (2006). Phylogenetic relationships of the lamprologine cichlid genus *Lepidiolamprologus* (Teleostei: Perciformes) based on mitochondrial and nuclear sequences, suggesting introgressive hybridization. *Molecular Phylogenetics and Evolution*.

[38] Day JJ, Santini S, Garcia-Moreno J (2007). Phylogenetic relationships of the Lake Tanganyika cichlid tribe lamprologini: the story from mitochondrial DNA. *Molecular Phylogenetics and Evolution*.

[39] Koblmüller S, Duftner N, Sefc KM (2007). Reticulate phylogeny of gastropod-shell-breeding cichlids from Lake Tanganyika—the result of repeated introgressive hybridization. *BMC Evolutionary Biology*.

[40] Koblmüller S, Egger B, Sturmbauer C, Sefc KM (2010). Rapid radiation, ancient incomplete lineage sorting and ancient hybridization in the endemic Lake Tanganyika cichlid tribe tropheini. *Molecular Phylogenetics and Evolution*.

[41] Nevado B, Koblmüller S, Sturmbauer C, Snoeks J, Usano-Alemany J, Verheyen E (2009). Complete mitochondrial DNA replacement in a Lake Tanganyika cichlid fish. *Molecular Ecology*.

[42] Sturmbauer C, Salzburger W, Duftner N, Schelly R, Koblmüller S (2010). Evolutionary history of the Lake Tanganyika cichlid tribe lamprologini (Teleostei: Perciformes) derived from mitochondrial and nuclear DNA data. *Molecular Phylogenetics and Evolution*.

[43] Boulenger GA (1895–1896). Report on the collection of fishes made by Mr. J. E. S. Moore in Lake Tanganyika during his expedition. *Transactions of the Zoological Society of London*.

[44] Poll M (1946). Révision de la faune ichthyologique du lac Tanganika. *Annales du Musée du Congo Belge Série 1*.

